# Oosporein Produced by Root Endophytic *Chaetomium cupreum* Promotes the Growth of Host Plant, *Miscanthus sinensis*, under Aluminum Stress at the Appropriate Concentration

**DOI:** 10.3390/plants12010036

**Published:** 2022-12-21

**Authors:** Toshikatsu Haruma, Kohei Doyama, Xingyan Lu, Takahiko Arima, Toshifumi Igarashi, Shingo Tomiyama, Keiko Yamaji

**Affiliations:** 1 Division of Sustainable Resources Engineering, Faculty of Engineering, Hokkaido University, Kita 13, Nishi 8, Kita-ku, Sapporo, Hokkaido 060-8628, Japan; 2 Graduate School of Life and Environmental Sciences, University of Tsukuba, 1-1-1, Tennoudai, Tsukuba, Ibaraki 305-8572, Japan

**Keywords:** *Miscanthus sinensis*, *Chaetomium cupreum*, oosporein, Al tolerance, root endophyte, mine site

## Abstract

*Chaetomium cupreum*, a root endophyte in *Miscanthus sinensis*, enhances Al tolerance in *M. sinensis* by changing aluminum (Al) localization and the production of a siderophore, oosporein, which chelates Al for detoxification. Oosporein has various functions, including insecticidal activity, phytotoxicity, antifungal activity, and a siderophore. In our study, we focused on the detoxification effect of oosporein as a siderophore and on the growth of *M. sinensis* under Al exposure. In addition, the phytotoxicity of oosporein to *M. sinensis* was confirmed to compare with those in *Lactuca sativa* and *Oryza sativa* as control plants. Under Al stress, oosporein promoted plant growth in *M. sinensis* seedlings at 10 ppm, which was the same concentration as that detected in *M. sinensis* roots infected with *C. cupreum* in our previous study. Oosporein also showed low phytotoxicity to *M. sinensis* compared with *L. sativa* at even high concentrations of oosporein. These results suggest that the concentration of oosporein in *M. sinensis* roots would be maintained at the appropriate concentration to detoxify Al and would promote *M. sinensis* growth under Al stress, although oosporein would show low phytotoxicity to the natural host plant, *M. sinensis*, compared with the non-host plant, *L. sativa*.

## 1. Introduction

Aluminum (Al) is ubiquitously present in soils as a major component, and has been recognized as a toxic factor in acidic soils during the last century [1]. In acidic environments, such as mine sites, Al dissolves in soil solution as Al^3+^ [2], which inhibits plant growth [3,4,5] and decreases essential nutrient concentrations in plants [4]. *Miscanthus sinensis*, a common perennial plant in Japan, is commonly observed at various mine sites. In addition to the mine site, *M. sinensis* can dominate disturbed ecosystems, such as volcanic areas with acid-sulfate soils [6]. A previous report [7] showed that *M. sinensis* can accumulate high concentrations of Al in its roots and grow without toxic Al symptoms. Our previous research clarified that *M. sinensis* growing at mine sites can tolerate Al toxicity via the functions of root endophytes [8,9].

Root endophytic fungi can enhance the tolerance of host plants to environmental stresses, such as salt, drought, herbivorous pathogens, and metals [10]. Terrestrial plants are hosts to fungal root endophytes [11], which are defined as “bacteria and fungi that live plant tissues without causing disease” [12]. Although root endophytic fungi do not necessarily provide plants with beneficial effects, unlike mycorrhiza fungi [13], root endophytes help the host plants survive severe environmental stresses [10]. Our previous study showed that the root endophytic *Chaetomium cupreum* isolated from *M. sinensis* enhanced Al tolerance in *M. sinensis* by (1) altering Al localization in the roots of *M. sinensis* into the cell walls of the epidermis, endodermis, and stele, which are harmless tissues in roots, and (2) producing oosporein, which detoxifies Al by chelation [8]. Our previous study also showed that oosporein produced by the root endophytic *C. cupreum* has a higher stability constant with Al than secondary metabolites (chlorogenic acid, citric acid, and malic acid), which were produced by *M. sinensis* to detoxify Al. Compounds with higher stability constants with Al chelate Al more strongly to detoxify Al efficiently compared with those with lower stability constants. Therefore, oosporein could play a crucial role in Al tolerance in *M. sinensis* [8].

Siderophores are known to promote plant growth via promoting the uptake of Fe, which is an important mineral in plant nutrition [14]. Initially, siderophores were defriend as low-molecular-weight and Fe-specific ligands [14]. Recently, root endophytic fungi have been known to produce siderophores, which chelate various metals such as Al, Cd, and Cu, in addition to Fe [15]. Therefore, siderophores produced by endophytes detoxify harmful metals, resulting in an increased tolerance to harmful metals in host plants as well [16,17]. Oosporein, which was isolated as a siderophore in our previous study [18], was first isolated from a culture solution of *Oospora colorans* [19], and recognized as an insecticidal compound produced by the insect pathogenic fungus, *Beauveria bassiana* [20]. Afterward, oosporein was also recognized as a phytotoxic compound in *Nicotiana tabacum* [21] and as an antifungal compound [22]. A siderophore stimulated the metabolism of *Arabidopsis thaliana* to defend against pathogenic bacteria [23]. In our previous study [18], infection with *C. cupreum* producing oosporein enhanced *M. sinensis* growth under Al stress; however, we could not clarify whether oosporein itself could increase Al tolerance and promote *M. sinensis* growth.

In this study, we focused on the function of oosporein in *M. sinensis*. The purpose of this study was to clarify whether oosporein itself could enhance Al tolerance in *M. sinensis* to promote plant growth and element uptake under Al stress. Additionally, we clarified the phytotoxicity of oosporein to *M. sinensis* compared with that in *Lactuca sativa* and *Oryza sativa*, which is an ordinal model plant to assay phytotoxicity, and a typical and agriculturally important plant within *Poaceae* family, similar to *M. sinensis* in Japan, respectively. Finally, we discuss the function of oosporein under Al stress and the phytotoxicity to *M. sinensis*.

## 2. Results

### 2.1. Effect of Oosporein on Growth and Elements Uptake of M. sinensis Incubated under Al Stress

**Figure 1:**
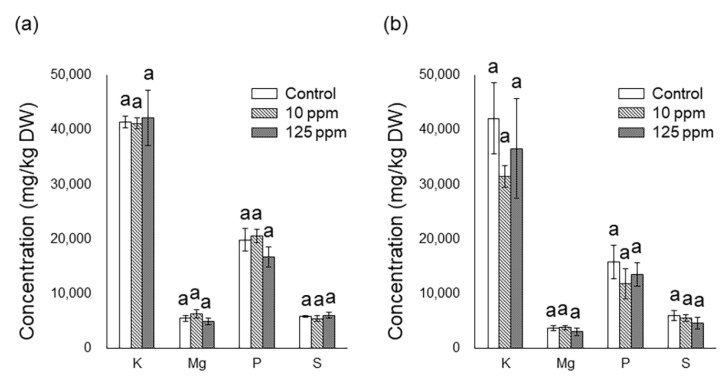
Nutrient element concentrations in *Miscanthus sinensis* seedlings after incubation in nutrient solution containing 100 µM Al after oosporein treatment. (**a**) Concentration in aboveground parts, and (**b**) concentration in roots. After incubation of 3 h in 0, 10, or 125 ppm solutions of oosporein, the seedlings were transferred to 1/10 Hoagland solution containing 100 µM Al and incubated for 30 days. Data of each bar followed by the same letter (a) are not significantly different among treatment in ANOVA comparisons and post hoc Tukey HSD at *p* < 0.05 (*n* = 4). Error bars represent ± SE.

**Figure 2:**
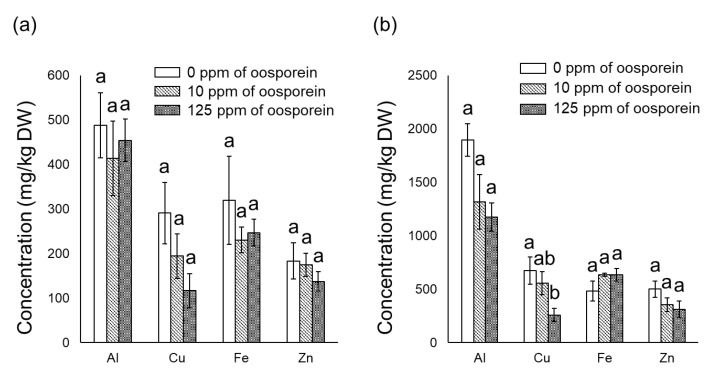
Al and heavy metal concentrations in *Miscanthus sinensis* seedlings after incubation in nutrient solution containing 100 µM Al after oosporein treatment. (**a**) Concentration in aboveground parts, and (**b**) concentration in roots. After incubation of 3 h in 0, 10, or 125 ppm solutions of oosporein, the seedlings were transferred to 1/10 Hoagland solution containing 100 µM Al and incubated for 30 days. Different letters (a and b) indicate a statistically significant difference among treatment in ANOVA comparisons and post hoc Tukey HSD at *p* < 0.05 (*n* = 4). Error bars represent ± SE.

After incubation in 1/10 Hoagland solution containing 100 µM Al, the fresh weight (FW) and dry weight (DW) of roots soaked in 10 ppm of oosporein were significantly increased (Table 1). Oosporein did not have any effect on the uptake of nutrient elements in the aboveground parts and roots (Figure 1). Oosporein at 125 ppm markedly decreased the Cu concentration in the roots (Figure 2b) and seemed to reduce the Al concentration (*p* = 0.056) (Figure 2b).

### 2.2. Effects of Oosporein on Growth of L. sativa, O. sativa, and M. sinensis Seedlings in Growth Inhibition Test

**Figure 3:**
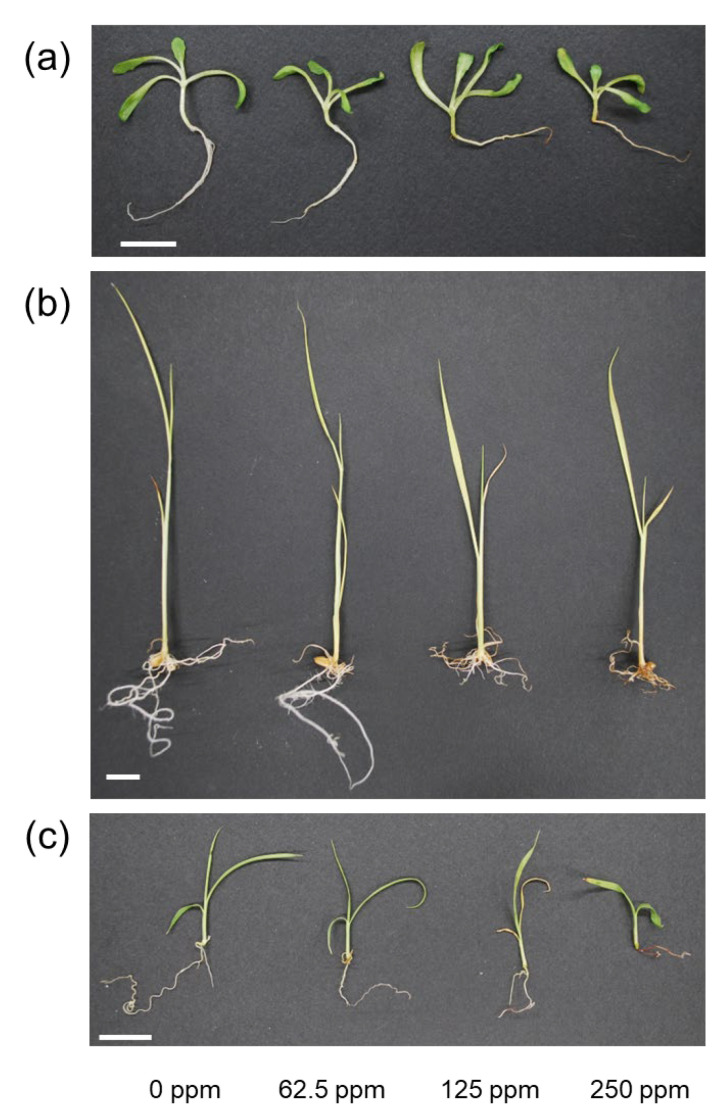
Phytotoxicity of oosporein to seedling growths of *Lactuca sativa*, *Oryza sativa*, and *Miscanthus sinensis*. (**a**–**c**) show seedlings of *L. sativa*, *O. sativa*, and *M. sinensis* after incubation in oosporein solution at each concentration for 10 days, respectively. Scale bars represent 10 mm.

In the growth inhibition test using *L. sativa* as a control plant, oosporein at 125 and 250 ppm significantly inhibited root length and water content of the aboveground parts (Table 2). At 250 ppm, oosporein markedly decreased the FW of aboveground parts and roots, as well as the water content of the roots (Table 2). Oosporein at 125 and 250 ppm caused browning of the roots of *L. sativa* (Figure 3a). When *O. sativa* was used as a control plant in the *Poaceae* family, 250 ppm of oosporein significantly increased the FW of the aboveground parts and roots (Table 3). At 125 ppm, oosporein significantly increased the water content of the aboveground parts (Table 3). The water content of roots significantly increased after 125 and 250 ppm of oosporein treatments (Table 3). However, oosporein at 125 and 250 ppm caused the roots of *O. sativa* to become brown (Figure 3b). In *M. sinensis*, oosporein significantly decreased the FW of aboveground parts by 125 ppm of oosporein (Table 4). The water content of the aboveground parts decreased by 125 and 250 ppm of oosporein (Table 4). Oosporein at 125 and 250 ppm caused the roots of *M. sinensis* to turn brown (Figure 3c).

## 3. Discussion

Recently, it was demonstrated that a siderophore promote plant growth via alleviating metal-induced oxidative stress in plants in contaminated soil [24]. Oosporein produced by the root endophytic *C. cupreum*, which was isolated from *M. sinensis*, has a higher stability constant with Al; therefore, oosporein could act a crucial role in Al tolerance in *M. sinensis* [8]. In our study, 10 ppm of oosporein could alleviate Al stress and enhance the growth of *M. sinensis* (Table 1). The concentration of oosporein at 10 ppm was the same as that detected in our previous inoculation test using the root endophytic *C. cupreum* and *M. sinensis* [18]. These results indicate that 10 ppm of oosporein would be appropriate for enhancing growth and Al tolerance in *M. sinensis*. In contrast, 125 ppm of oosporein significantly decreased the root FW of *M. sinensis* compared with 10 ppm of oosporein (Table 1). Therefore, 125 ppm of oosporein would be excessive for *M. sinensis* growing under Al stress.

Siderophores are defined as relatively low-molecular-weight compounds capable of chelating Fe [25] and various metals, including Al [10,26,27,28]. Oosporein chelates Al [18] and is a kind of catechol-type siderophores, which have hydroxyl groups to chelate Cu and Fe [29]. Therefore, we analyzed nutrient elements and Al uptakes in *M. sinensis* to clarify the factors for growth enhancement by oosporein exposure. Although a siderophore promoted the uptake of Fe [15], the concentrations of K, Mg, P, S, Fe and Zn in the aboveground parts and roots of *M. sinensis* were not increased at any concentration of oosporein (Figure 1 and Figure 2). Concentrations of Cu in roots were significantly decreased at 125 ppm of oosporein, and Al concentrations in roots seemed to decrease with increasing oosporein concentrations (Figure 2b), which suggested that oosporein might chelate Cu and Al outside the roots, resulting in the suppression of Cu and Al uptake. These results are consistent with those of an inoculation test using *C. cupreum* and *M. sinensis* [18], which indicated that oosporein produced by *C. cupreum* would enhance *M. sinensis* to adapt to Al and heavy metal stress.

In relationships between root endophytes and host plants, the fungal phytotoxicity and plant defense system should be balanced to enhance various environmental stresses in plants by root endophytes [30]. For *Chaetomium* species, it is crucial to assess the phytotoxicity of their productions because *Chaetomium* species produce various chemical compounds, which are used as biological control against plant diseases [31,32,33,34]. Siderophores have been reported to exhibit various bioactivities, such as antifungal and antibacterial activities [35,36,37]. Among siderophores, oosporein has also been reported to exhibit antifungal activity [38,39] and toxicity to insects [40,41]. Our study clarified that oosporein at 125 ppm showed phytotoxicity as evidenced by browning of roots of *L. sativa*, *O. sativa*, and *M. sinensis*. Oosporein significantly decreased the root length of *L. sativa* by 40% and 50% at 125 and 250 ppm, respectively (Table 2). In addition, 250 ppm of oosporein remarkably reduced the FW of the aboveground parts and roots by 20% and 35%, respectively (Table 2). Although oosporein significantly decreased the FW of aboveground parts of *M. sinensis* by 25% at 125 ppm, the root length of *M. sinensis* were not decreased at any oosporein concentration (Table 4). These results suggest that oosporein has low phytotoxicity to *M. sinensis*, a natural host plant, compared with *L. sativa*. In the case of *O. sativa*, oosporein did not significantly inhibit growth (Table 3). These results suggest that *Poaceae* family plants, which *O. sativa* and *M. sinensis* belong to, might have a certain tolerance to oosporein compared with other families of plants such as *L. sativa*. Antifungal compounds such as patulin, citrinin, frequentin, and palitantin were isolated from three seed-epiphytic *Penicillium* strains of *Picea glehnii* [42]. Patulin, citrinin, and frequentin significantly inhibited the growth of *L. sativa* seedlings. However, these compounds did not show phytotoxicity to *P. glehnii,* indicating that this natural host plant, *P. glehnii*, has a certain tolerance against these compounds produced by seed-epiphytic *Penicillium* fungi. Our results also indicate that oosporein produced by root endophytic *C. cupreum* in the rhizosphere of *M. sinensis* would show low phytotoxicity to the natural host plant, *M. sinensis*, compared with the non-host plant, *L. sativa*.

## 4. Materials and Methods

### 4.1. Effect of Oosporein on the Growth and Elements Concentration of M. sinensis Seedlings under Al Stress

#### 4.1.1. Isolation of Oosporein from Culture Solution of *C. cupreum*

Oosporein was isolated from the mycelial solution of *C. cupreum* according to [18]. *Chaetomium cupreum* was grown on 1% malt extract agar (1% MA) for seven days at 23 °C in the dark to obtain mycelial disks (5.5 mm i.d.) at the edge of the mycelium. Twenty mycelial disks of *C. cupreum* were inoculated in a 300 mL Erlenmeyer flask containing 100 mL of 1% malt extract liquid medium with shaking at 23 °C in the dark for 12 days. After inoculation, the mycelial disks were removed by filtration using No. 6 filter paper (Advantec, Tokyo, Japan). The culture filtrate (900 mL, 7.27 g dry weight (DW)) was concentrated to 150 mL and extracted three times by ethyl acetate (50 mL each). After drying over Na_2_SO_4_, followed by drying in vacuo at 40 °C, an organic layer (237 mg) was obtained. Residuals in the organic layer were crystallized using cold ethanol and crystalline powder (red amorphous). The crystalline powder of oosporein was dried in vacuo at 25 °C. The identification of oosporein was followed by the procedures of [18].

#### 4.1.2. Growth Condition

*Miscanthus sinensis* seeds were collected at the Hitachi mine in November 2018 and stored at 4 °C until use. Seeds were sterilized as described by [8]. The seeds were surface-sterilized with 70% ethanol for 1 min, 7.5% hydrogen peroxide solution for 5 min, and 70% ethanol for 1 min. The seeds were then rinsed twice with sterilized water. Sterilized seeds were incubated on 1/3 Hoagland medium containing 1.5% agar (14 h light at 25 °C/10 h dark at 20 °C) in a growth chamber (NK Systems LP-100S, Nippon Medical & Instruments Co., Osaka, Japan) to a 2.0-leaf stage. Seedlings at the 2.0-leaf stage were used in the test. In our previous study [18], an inoculation test using *C. cupreum* and *M. sinensis* showed that *C. cupreum* produced oosporein in *M. sinensis* roots at 10 µg/g fresh weight (10 ppm FW). According to our previous study [18], 0, 10, and 125 ppm solutions of oosporein dissolved in methanol (2 mL each) were added to filter paper in a glass dish (90 mm i.d.). An oosporein concentration of 125 ppm was used as an excessive concentration for *M. sinensis*. The dishes were kept in vacuo to remove the methanol. Sterilized deionized water (2 mL each) was added to the dish and two seedlings of *M. sinensis* were transferred. After incubation of 3 h, the seedlings were transferred to 1/10 Hoagland solution containing 100 µmol/L Al (pH 4.0, 0.1 M HCl) and incubated for 30 days. At each concentration of oosporein, one *M. sinensis* seedling was used for measuring root length, FW of the aboveground parts and roots, and DW of the aboveground parts and roots. Each test was repeated four times.

#### 4.1.3. Concentration of Nutrient Elements, Al, and Heavy Metals in Roots of *M. sinensis*

The seedlings were separated into aboveground parts and roots. The roots were washed with 0.5 mmol/L CaCl_2_ (20 min × 3 times) to remove Al on their surface and rinsed with deionized water. The aboveground parts and roots were dried at 80 °C for 48 h and pyrolyzed with HNO_3_ at 140 °C. We quantified the nutrient elements (K, Mg, P, and S), Al, and heavy metals (Cu, Fe, and Zn), which were contained in culture solution, of seedlings using inductively coupled plasma optical emission spectrometry (ICP-OES; Optima 7300 V, PerkinElmer, Waltham, MA, USA).

### 4.2. Growth Inhibition Test Using L. sativa, O. sativa, and M. sinensis Seedlings by Oosporein

In the aforementioned experiment (see Section 4.1.2.), 125 ppm of oosporein did not enhance the growth of *M. sinensis* (Table 1), suggesting that 125 ppm might show low phytotoxicity to *M. sinensis*. Sterilized seedlings of the 2.0-leaf stage *M. sinensis* were prepared as described above. *Lactuca sativa* (Legacy) and *O. sativa* (Nipponbare) were used as control plants for the growth inhibition test. *Lactuca sativa* seeds were purchased from Kuragi Co. Ltd. (Mie, Japan). Non-sterilized seeds were incubated on 1/3 Hoagland medium containing 1.5% agar (14 h light at 25 °C/10 h dark at 20 °C) in a growth chamber. Seedlings were used for the growth inhibition test for five days after germination. *Oryza sativa* seeds were purchased from the Nouken Corporation (Kyoto, Japan). Non-sterilized seeds were germinated as described by [43]. The seeds were soaked in distilled water and kept in the dark at 30 °C for three days. The germinated seedlings were transferred to 1/3 Hoagland medium containing 0.5% agar and incubated (14 h light at 25 °C/10 h dark at 20 °C) in a growth chamber to the 2.5-leaf stage. Seedlings at the 2.5-leaf stage were used for the growth inhibition tests that were conducted according to the results of aforementioned experiment (see Section 4.1.2.) and [42]. A 0, 62.5, 125, and 250 ppm solution of oosporein dissolved in methanol (2 mL each) was added to the filter paper in a glass dish (90 mm i.d.). The dishes were kept in vacuo to remove the methanol. Sterilized deionized water (2 mL each) was added to the dish and five seedlings of each plant species were transferred. Each test was performed in triplicate. The seedlings were incubated for 10 days (14 h light at 25 °C/10 h dark at 20 °C) in the growth chamber to avoid nutrient deficiency for plants growth. Root length, FW of the aboveground parts and roots, and DW of the aboveground parts and roots were measured.

### 4.3. Statistical Analysis

Statistical analysis was conducted using the SPSS software for Windows (ver. 26.0.0.1, IBM, Armonk, NY, USA). Differences in seedling growth and Al concentrations were evaluated using a one-factor ANOVA test (Tukey HSD). Differences were considered statistically significant at *p* < 0.05.

## 5. Conclusions

In our study, oosporein itself promoted *M. sinensis* growth under Al stress at 10 ppm, which was the same concentration as that detected in *M. sinensis* roots infected with *C. cupreum* [18], suggesting that the appropriate concentration of oosporein was maintained to promote the growth of *M. sinensis* under Al stress. The growth inhibition test indicated that oosporein showed low phytotoxicity to a natural host plant, *M. sinensis*, compared with a non-host plant, *L. sativa*. To enhance tolerance to various environmental stresses in plants by root endophytes, it is crucial to maintain the balance between host plant defense and root endophyte virulence [30]. Our research suggests that oosporein at the appropriate concentration maintains the balance of the interaction between *M. sinensis* and the root endophytic *C. cupreum*, resulting in increased Al tolerance in *M. sinensis*, which could grow under Al stress such as mine sites.

## Figures and Tables

**Table 1:** Seedling growth of *Miscanthus sinensis* in liquid culture containing 100 µM Al after oosporein treatment.

Treatment of Oosporein	Root Length (cm)	Aboveground Parts FW (mg)	Roots FW (mg)	Aboveground Parts DW (mg)	Roots DW (mg)	Water Content in Aboveground Parts (%)	Water Content in Roots (%)
0 ppm	8.87 ± 1.41 a	8.71 ± 0.57 a	3.18 ± 0.15 a	0.79 ± 0.06 a	0.23 ± 0.01 a	90.95 ± 0.65 a	92.66 ± 0.72 a
10 ppm	10.55 ± 0.50 a	8.96 ± 0.33 a	4.07 ± 0.06 b	0.83 ± 0.06 a	0.33 ± 0.02 b	90.76 ± 0.41 a	91.84 ± 0.54 a
125 ppm	11.60 ± 2.15 a	8.39 ± 1.09 a	3.51 ± 0.18 a	0.87 ± 0.12 a	0.29 ± 0.03 ab	89.67 ± 0.62 a	91.55 ± 1.16 a

After incubation of 3 h in 0, 10, or 125 ppm solutions of oosporein, the seedlings were transferred to 1/10 Hoagland solution containing 100 µM Al and incubated for 30 days. FW: fresh weight. DW: dry weight. Different letters (a,b) indicate a statistically significant difference among treatments in ANOVA comparisons and post hoc Tukey HSD at *p* < 0.05 (*n* = 4).

**Table 2:** Seedling growth of *Lactuca sativa* in growth inhibition test by oosporein.

Treatment of Oosporein	Root Length (cm)	Aboveground Parts FW (mg)	Roots FW (mg)	Aboveground Parts DW (mg)	Roots DW (mg)	Water Content in Aboveground Parts (%)	Water Content in Roots (%)
0 ppm	8.15 ± 0.43 a	24.96 ± 1.18 a	5.83 ± 0.30 a	1.04 ± 0.05 a	0.26 ± 0.01 a	95.82 ± 0.06 a	95.44 ± 0.27 a
62.5 ppm	6.18 ± 0.75 ab	23.45 ± 0.90 ab	5.21 ± 0.34 ab	1.03 ± 0.03 a	0.23 ± 0.02 a	95.60 ± 0.07 ab	95.42 ± 0.37 a
125 ppm	4.99 ± 0.55 bc	23.99 ± 1.24 ab	4.91 ± 0.47 ab	1.08 ± 0.05 a	0.25 ± 0.02 a	95.47 ± 0.07 b	94.78 ± 0.32 ab
250 ppm	4.06 ± 0.40 c	20.16 ± 1.62 b	3.95 ± 0.41 b	1.03 ± 0.08 a	0.25 ± 0.02 a	94.85 ± 0.09 c	93.00 ± 0.86 b

The seedlings were incubated in oosporein solution at each concentration for 10 days. FW: fresh weight. DW: dry weight. Different letters (a–c) indicate a statistically significant difference among treatments in ANOVA comparisons and post hoc Tukey HSD at *p* < 0.05 (*n* = 15). Mean values are shown with ±SE.

**Table 3:** Seedling growth of *Oryza sativa* in growth inhibition test by oosporein.

Treatment of Oosporein	Root Length (cm)	Aboveground Parts FW (mg)	Roots FW (mg)	Aboveground Parts DW (mg)	Roots DW (mg)	Water Content in Aboveground Parts (%)	Water Content in Roots (%)
0 ppm	47.14 ± 2.01 a	35.46 ± 1.54 ab	15.18 ± 1.37 a	8.80 ± 0.35 a	3.13 ± 0.17 a	74.83 ± 1.07 a	76.14 ± 3.54 a
62.5 ppm	42.06 ± 3.12 a	34.04 ± 1.32 a	15.32 ± 1.22 a	8.50 ± 0.22 a	3.02 ± 0.16 a	74.51 ± 1.18 a	79.47 ± 1.19 ab
125 ppm	37.01 ± 3.48 a	41.20 ± 1.90 bc	17.89 ± 1.95 ab	8.04 ± 0.42 a	2.50 ± 0.25 a	80.43 ± 0.69 b	85.65 ± 0.54 bc
250 ppm	41.41 ± 3.12 a	42.58 ± 2.22 c	22.57 ± 1.76 b	9.23 ± 0.40 a	3.02 ± 0.21 a	77.95 ± 0.80 ab	86.22 ± 0.68 c

The seedlings were incubated in oosporein solution at each concentration for 10 days. FW: fresh weight. DW: dry weight. Different letters (a–c) indicate a statistically significant difference among treatments in ANOVA comparisons and post hoc Tukey HSD at *p* < 0.05 (*n* = 15). Mean values are shown with ± SE.

**Table 4:** Seedling growth of *Miscanthus sinensis* in growth inhibition test by oosporein.

Treatment of Oosporein	Root Length (cm)	Aboveground Parts FW (mg)	Roots FW (mg)	Aboveground Parts DW (mg)	Roots DW (mg)	Water Content in Aboveground Parts (%)	Water Content in Roots (%)
0 ppm	6.13 ± 0.71 a	5.11 ± 0.27 a	1.73 ± 0.27 a	0.63 ± 0.04 a	0.19 ± 0.02 a	87.62 ± 0.39 a	88.00 ± 1.12 ab
62.5 ppm	4.65 ± 0.50 a	4.76 ± 0.28 ab	1.09 ± 0.11 a	0.63 ± 0.04 a	0.16 ± 0.02 a	86.69 ± 0.51 ab	84.67 ± 1.71 a
125 ppm	5.25 ± 0.82 a	3.85 ± 0.33 b	1.40 ± 0.15 a	0.57 ± 0.05 a	0.20 ± 0.03 a	84.86 ± 0.70 b	85.68 ± 1.32 ab
250 ppm	4.34 ± 0.65 a	4.33 ± 0.35 ab	1.26 ± 0.16 a	0.64 ± 0.06 a	0.14 ± 0.02 a	85.25 ± 0.54 b	89.49 ± 0.81 b

The seedlings were incubated in oosporein solution at each concentration for 10 days. FW: fresh weight. DW: dry weight. Different letters (a,b) indicate a statistically significant difference among treatments in ANOVA comparisons and post hoc Tukey HSD at *p* < 0.05 (*n* = 15). Mean values are shown with ± SE.

## Data Availability

The data are not publicly available due to privacy.
